# Rab32 uses its effector reticulon 3L to trigger autophagic degradation of mitochondria-associated membrane (MAM) proteins

**DOI:** 10.1186/s13062-021-00311-9

**Published:** 2021-11-07

**Authors:** Maria Sol Herrera-Cruz, Megan C. Yap, Nasser Tahbaz, Keelie Phillips, Laurel Thomas, Gary Thomas, Thomas Simmen

**Affiliations:** 1 Department of Cell Biology, Faculty of Medicine and Dentistry, University of Alberta, Edmonton, AB T6G2H7 Canada; 2 Department of Microbiology and Molecular Genetics, University of Pittsburgh School of Medicine, Pittsburgh, PA 15219 USA

**Keywords:** Rab32, Mitochondria-associated membrane (MAM), Autophagy, ER-phagy

## Abstract

**Background:**

Rab32 is a small GTPase associated with multiple organelles but is particularly enriched at the endoplasmic reticulum (ER). Here, it controls targeting to mitochondria-ER contacts (MERCs), thus influencing composition of the mitochondria-associated membrane (MAM). Moreover, Rab32 regulates mitochondrial membrane dynamics via its effector dynamin-related protein 1 (Drp1). Rab32 has also been reported to induce autophagy, an essential pathway targeting intracellular components for their degradation. However, no autophagy-specific effectors have been identified for Rab32. Similarly, the identity of the intracellular membrane targeted by this small GTPase and the type of autophagy it induces are not known yet.

**Results:**

To investigate the target of autophagic degradation mediated by Rab32, we tested a large panel of organellar proteins. We found that a subset of MERC proteins, including the thioredoxin-related transmembrane protein TMX1, are specifically targeted for degradation in a Rab32-dependent manner. We also identified the long isoform of reticulon-3 (RTN3L), a known ER-phagy receptor, as a Rab32 effector.

**Conclusions:**

Rab32 promotes degradation of mitochondrial-proximal ER membranes through autophagy with the help of RTN3L. We propose to call this type of selective autophagy “MAM-phagy”.

**Supplementary Information:**

The online version contains supplementary material available at 10.1186/s13062-021-00311-9.

## Background

The ubiquitous small GTPase Rab32 is a multifunctional trafficking regulator that forms a Rab protein subfamily together with Rab29 and Rab38 [1]. Rab32 localizes to the cytosol, mitochondria, Golgi, and lysosome-related organelles (LROs) such as the melanosome and autophagosome [2]. This small GTPase has diverse functions at these organelles, but is particularly abundant on the ER [2]. Here, a large portion of Rab32 is a component of mitochondria-ER contacts (MERCs) [3]. MERCs can be isolated biochemically as mitochondria-associated membranes (MAMs) that house Ca^2+^ [4, 5] and lipid flux between the two organelles [6, 7]. On the mitochondrial membrane, Rab32 can anchor cAMP-dependent protein kinase (PKA), which in turn can inactivate the Rab32 effector dynamin-related protein 1 (Drp1) [3]. As a target of the unfolded protein response (UPR) [8], Rab32 moves Ca^2+^-regulatory ER proteins from MERCs to the cellular periphery, which results in increased Ca^2+^ transfer from the ER to mitochondria [3].

Despite the predominant localization of Rab32 to the ER, its activities also extend into the Golgi complex and LROs. Here, Rab32 interacts with sorting nexin 6 (SNX6) and mediates retrograde transport of leucine-rich repeat kinase 2 (LRRK2) to the Golgi [9]. In melanocytes, Rab32 and Rab38 control the delivery of tyrosinase to the melanosome [10] with the help of biogenesis of lysosome-related organelles complex 3 (BLOC-3) [11]. In hepatocytes, Rab32 has been detected on lysosomes, where it controls signaling by the mammalian target of rapamycin complex 1 (mTORC1) [12]. Taken together, these studies indicate Rab32 controls trafficking steps at multiple locations on the secretory pathway.

Interestingly, in non-melanocytic cells, Rab32 overexpression can increase autophagosome number [13]. This suggests Rab32 could activate macroautophagy, hereafter referred to as autophagy. One way this could occur is via interaction with an autophagy-related effector protein [14]. Indeed, several Rab proteins involved in autophagosome biogenesis interact with such autophagy effectors, including Rab37 and its effector Atg5 [15], as well as Rab11 and its effector, the TBC-domain family member 14 (TBC1D14) [16].

Some of these Rab effectors are autophagy receptors, proteins which link autophagic cargo to the autophagosomal membrane. Autophagy receptors allow cells to target specific organelles for selective autophagy. For example, the nuclear dot protein of 52 kDa (NDP52) is a mitochondrial autophagy receptor and effector of Rab35 during the process of mitophagy, the selective degradation of mitochondria (Minowa-Nozawa et al., [17]). Similarly, degradation of the ER, or ER-phagy, occurs through a variety of ER-specific autophagy receptors, including the protein called "cell cycle progression 1" (CCPG1), which specifically degrades peripheral ER during starvation [18]. Another example is Sec62, which degrades ER membranes enriched in chaperones following ER stress [19]. The protein called "family with sequence similarity 134 member B" (FAM134B) is instead involved in the degradation of ER sheets [20] while reticulon-3 (RTN3) targets ER tubules [21].

Consistent with a role for Rab32 in autophagy, syntaxin-17 (STX17), a soluble N-ethylmaleimide-sensitive factor attachment protein receptor (SNARE) and effector of Rab32, is involved in multiple steps in autophagy [22]. Firstly, STX17 acts to recruit Atg14L to MERCs during autophagosome biogenesis [23]. Moreover, STX17 can control the assembly of the mammalian pre-autophagosomal structure (mPAS) upon interaction with Atg13 [24]. It also localizes to fully closed autophagosomes and mediates their fusion to lysosomes (Itakura et al., 2012).

The multitude of intracellular locations and functions reported for Rab32 have so far prevented the identification of a clear-cut mechanistic role for Rab32 in autophagy. Our results address this deficiency and show activation of Rab32 induces selective autophagy of MERCs via interaction with the long form of the ER-phagy receptor reticulon-3 (RTN3L), which acts as an effector of GTP-bound Rab32.

## Results

### Rab32 promotes autophagy

**Fig. 1:**
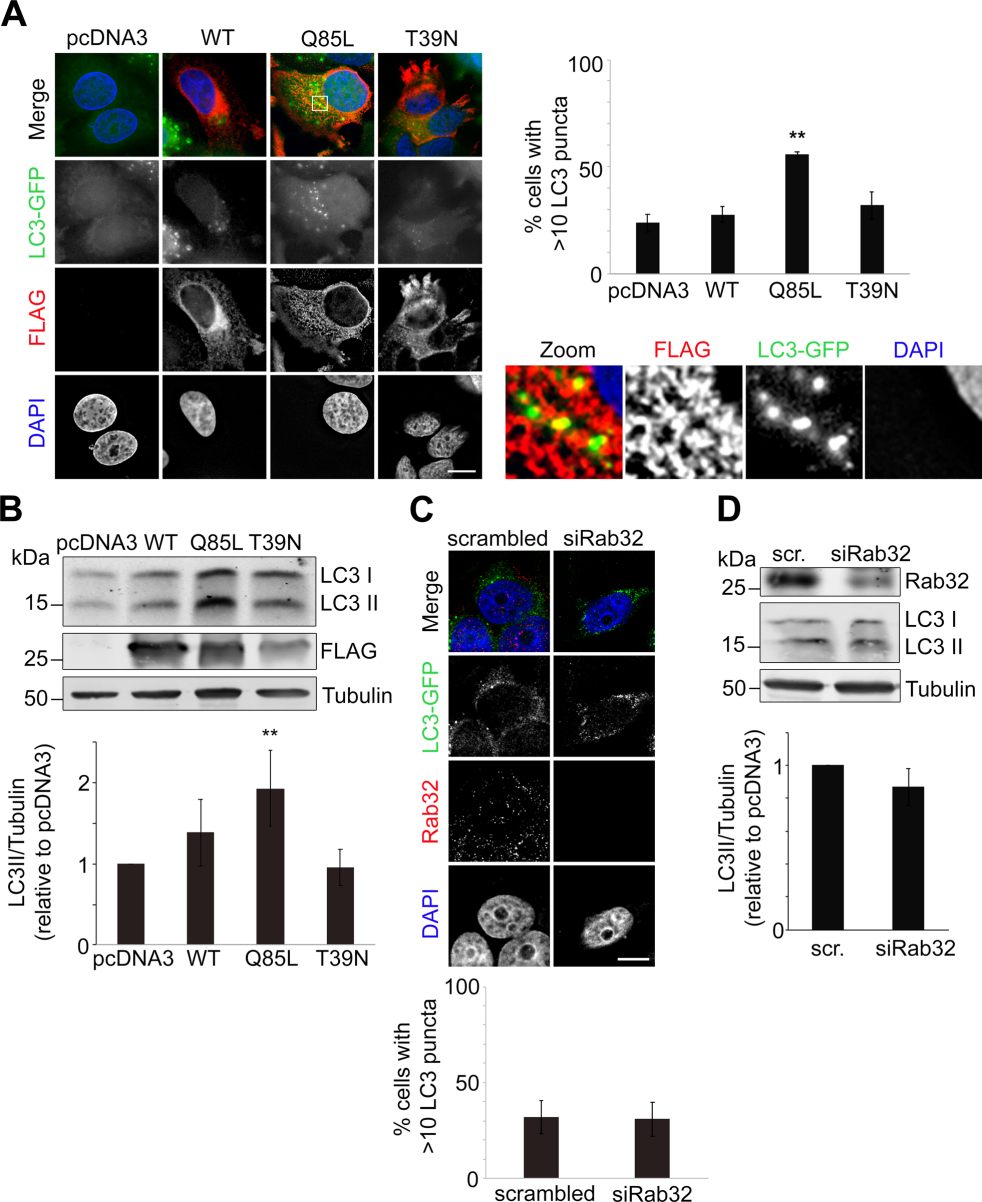
Active Rab32 Promotes Autophagy. **A** Representative immunofluorescence images of MCF7 cells stably expressing LC3-GFP and transfected with FLAG-tagged wild type (WT), dominant active (Q85L) or dominant negative (T39N) Rab32, probed for FLAG-tagged Rab32 (red) and nuclei (DAPI, blue) in parallel. Bar indicates 15 µm. Quantification of cells with > 10 LC3 puncta, indicating increased autophagy, were expressed as a percent of total cells counted. ≥ 100 cells per condition were counted in each (n = 3) independent experiment **p ≤ 0.001. Inset shows magnification of a representative area, evidencing overlap between Rab32 and LC3-GFP. **B** Immunoblot of MCF7 cells transfected with pcDNA3 vector as a control, or with Rab32 plasmids as indicated. Blots were probed for LC3, FLAG and Tubulin as a loading control. Densitometric analysis of LC3II/Tubulin on the bottom. n = 3 **p ≤ 0.001. **C** Representative immunofluorescence images of MCF7 cells stably expressing LC3-GFP with control siRNA or siRNA against Rab32, probed for endogenous Rab32 (red) and nuclei (DAPI, blue) in parallel. Bar indicates 10 µm. Quantification of cells with > 10 LC3 puncta, indicating increased autophagy, were expressed as percent of total cells counted. ≥ 100 cells per condition were counted in each (n = 3) independent experiment. *p* > 0.05. **D** Immunoblot of MCF7 cells with control siRNA or siRab32 probed for endogenous Rab32 and LC3. Densitometric analysis of LC3II/Tubulin on the bottom. n = 3, *p* > 0.05

Rab32 has been implicated in autophagy initiation for a long time since over-expressed Rab32 increases autophagosome number in COS-1 cells [13]. We first sought to confirm this finding by quantifying LC3 puncta generated from stably expressed GFP-labeled LC3 (GFP-LC3) in cells transfected with FLAG-tagged wild-type Rab32, dominant-active Rab32Q85L and dominant-negative Rab32T39N. As seen in Fig. 1A, about 20% of control cells had increased numbers of autophagosomes, thus establishing the baseline of autophagosome formation. Over-expression of both wild-type Rab32 and Rab32T39N did not significantly increase this percentage in MCF7 cells (Fig. 1A), unlike what had been reported in COS1 cells [13]. However, Rab32Q85L led to a doubling of these puncta in MCF7 cells like in COS-1 cells (Fig. 1A). Moreover, this active mutant of Rab32 partially co-localized with LC3, confirming previous findings that Rab32 is found on autophagosomes [13],Pei et al., 2015) (Fig. 1A). No effects were seen with the closely related Rab29 or Rab38 (Additional file 1: Fig. S1A). Next, we aimed to further characterize these results by probing for the status of LC3 and the autophagy receptor p62 within lysates. Consistent with our immunofluorescence results, only Rab32Q85L doubled baseline LC3 II amounts relative to tubulin (Fig. 1B). Rab32Q85L also caused a small reduction of p62, which is also consistent with an increase in autophagy (Additional file 1: Fig. S1B). Upon starvation, active Rab32Q85L did not increase the percentage of cells with abnormally high autophagosomes beyond the level observed with the pcDNA3 control (Additional file 1: Fig. S1C). Consistent with this, LC3 II levels were also unaffected with Rab32Q85L upon starvation (Additional file 1: Fig. S1D).

To test if the increase in LC3 clusters (Fig. 1A) and LC3 II levels (Fig. 1B) were not caused by a block in autophagosome degradation we incubated cells with Bafilomycin A1, a lysosomal degradation inhibitor. This caused a significant increase in LC3 II with Rab32Q85L (Additional file 1: Fig. S1D), suggesting active Rab32 did not increase autophagosome number by blocking autophagosome-lysosome fusion or by otherwise altering autophagic flux. Instead, these results strongly suggest Rab32Q85L induces autophagy.

Next, we decided to investigate if endogenous Rab32 is necessary for autophagy induction or autophagy progression. To approach this question, we knocked Rab32 down using RNAi. This resulted in a loss of the endogenous Rab32 fluorescence signal in MCF7 cells (Fig. 1C) and a reduction of the Western blot signal (Fig. 1D). However, we were neither able to detect changes in the number of GFP-labeled LC3 clusters, nor in the amount of LC3 II in lysates (Fig. 1C, D). This suggested that while Rab32 activity can trigger autophagy, Rab32 protein levels do not affect baseline autophagy induction or progression.

**Fig. 2:**
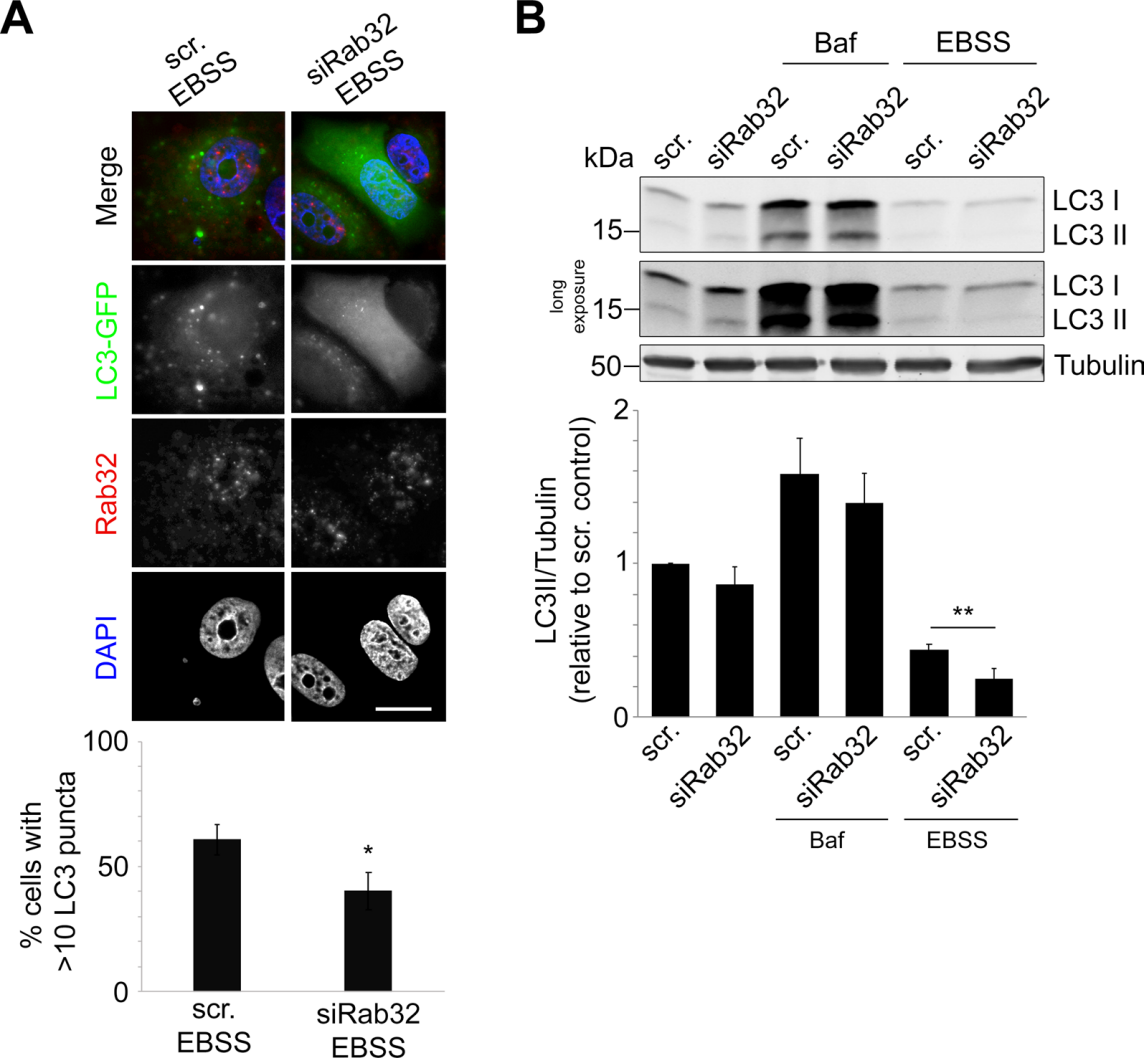
Rab32 Knockdown Impairs Starvation-Induced Autophagy.** A** Representative immunofluorescence images and quantification after a 2 h incubation with EBSS of MCF7 cells stably expressing LC3-GFP with control siRNA or siRNA against Rab32, probed for endogenous Rab32 (red) and nuclei (DAPI, blue) in parallel. Bar indicates 10 µm. Quantification of cells with > 10 LC3 puncta, indicating increased autophagy, were expressed as percent of total cells counted. ≥ 100 cells per condition were counted in each (n = 3) independent experiment. *p ≤ 0.05. **B** Immunoblot and densitometry analysis of MCF7 cells transfected with control siRNA or siRNA against Rab32, analyzed for LC3II/Tubulin after 4 h EBSS or 48 h 100 nM Bafilomycin A1 incubation. n = 3, **p ≤ 0.001

To further test this hypothesis, we exposed MCF7 cells with altered Rab32 activity and expression to starvation by incubating them for 2 h in Earle’s balanced salt solution (EBSS) that deprives cells of amino acids [25]. This medium led to an increase of GFP-labeled LC3 clusters, as expected (Fig. 2A). Specifically, the percentage of cells with abnormally high autophagosome numbers increased to roughly 60%, which was very close to that observed with active Rab32Q85L (Fig. 1A). However, there was a significant drop of autophagosome numbers upon Rab32 knockdown, suggesting endogenous Rab32 contributes to starvation-induced autophagy (Fig. 2A). Consistent with these results, starvation led to a further reduction of LC3 II in cells where we had knocked down Rab32, indicative of blocked autophagy (Fig. 2B). In contrast, incubation with Bafilomycin A1, an inhibitor of lysosomal degradation, did not cause significant differences in LC3 II between control and Rab32 knockdown cells (Fig. 2B). Together, these results suggest Rab32 activates an autophagic pathway, but not to levels beyond that of a short-term starvation stress.

### Rab32 interacts with reticulon-3L

**Fig. 3:**
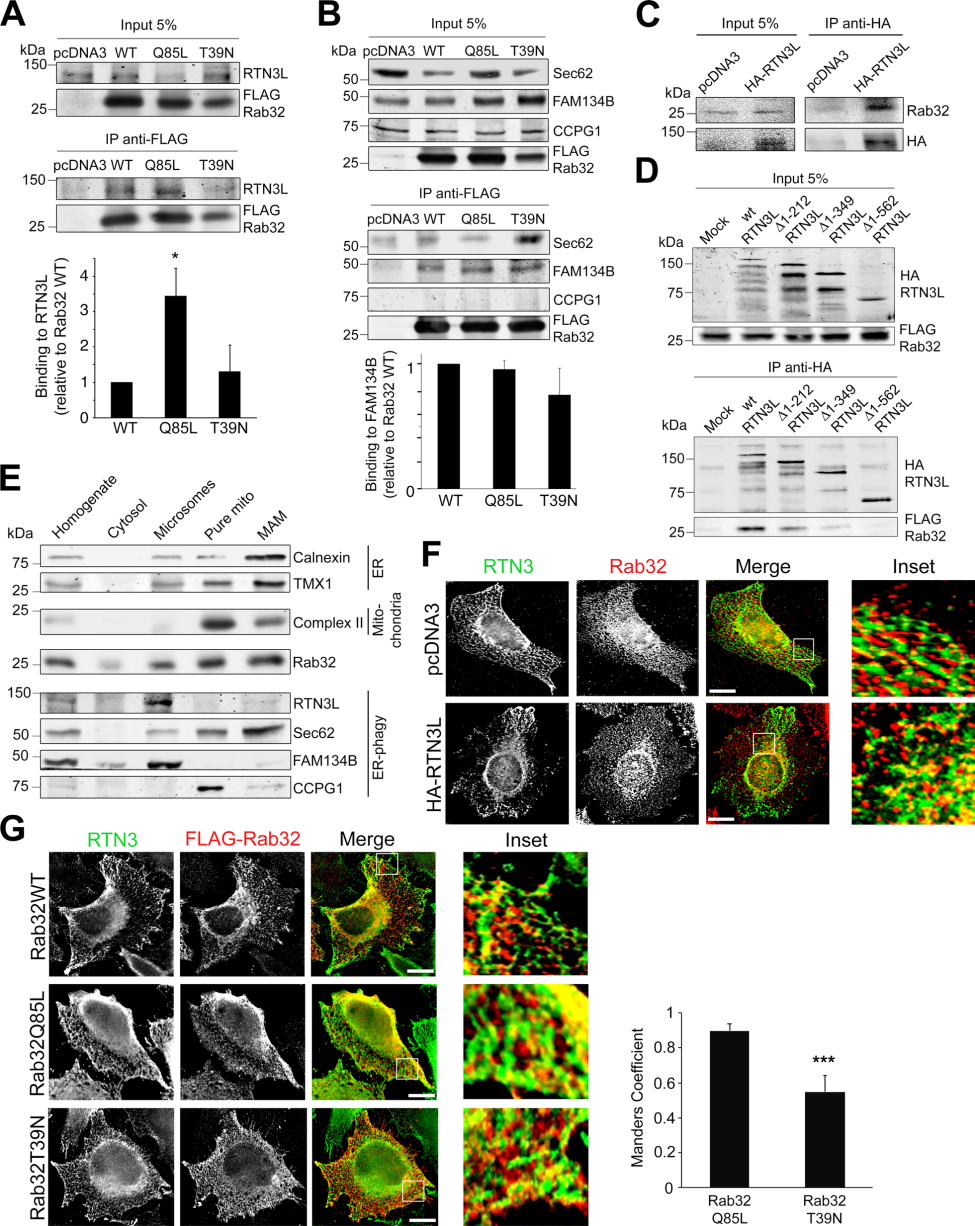
Rab32 interacts with the ER-phagy receptor Reticulon-3. **A** Co-immunoprecipitation of Rab32 constructs with endogenous RTN3L. MCF7 cells were transfected for 48 h with FLAG-tagged Rab32 constructs, followed by crosslinking, lysis and incubation with anti-FLAG antibodies. Immunoprecipitates were analyzed for anti-FLAG and co-immunoprecipitating endogenous long reticulon-3 (RTN3L). Densitometry of RTN3L binding normalized to respective input signals. n = 3, *p ≤ 0.05 **B** Co-immunoprecipitation of Rab32 constructs with endogenous Sec62, FAM134B and CCPG1. MCF7 cells were transfected for 48 h with FLAG-tagged Rab32 constructs, followed by crosslinking, lysis and incubation with anti-FLAG antibodies. Immunoprecipitates were analyzed for anti-FLAG and co-immunoprecipitating endogenous ER-phagy receptors. Densitometric FAM134B binding normalized to respective input signals. n = 3, *p* > 0.05. **C** Co-immunoprecipitation of endogenous Rab32 with HA-tagged RTN3L. MCF7 cells were transfected with HA-tagged RTN3L, followed by lysis and incubation with anti-HA antibodies. Immunoprecipitates were analyzed for anti-HA and co-immunoprecipitating endogenous Rab32. **D** Co-immunoprecipitation of FLAG-Rab32 constructs expressed from pcDNA3 with HA-tagged RTN3L and deletion mutants, expressed from pCEP4. HeLa cells were transfected for 48 h with FLAG-tagged Rab32 and HA-tagged RTN3L constructs, followed by crosslinking, lysis and incubation with anti-HA antibodies. Immunoprecipitates were analyzed on a 8–15% gradient gel/blot for anti-HA and co-immunoprecipitating FLAG-Rab32. The deletion mutants used were Δ1–212, Δ1–349, and Δ1–562. **E** Percoll fractionation of untransfected MCF7 cells for ER-phagy receptor proteins. Fractions stand for total homogenate, cytosol, microsomes, pure mitochondria and MAM (equal fractions loaded). **F** Immunofluorescence images of HeLa cells transfected with HA-tagged RTN3L (bottom) or empty pcDNA3 (top). Cells were incubated with antibodies against Rab32 and RTN3. Bar indicates 15 µm. Inset shows magnification of a representative area. **G** Immunofluorescence images of HeLa cells transfected with FLAG-tagged wild type (WT), dominant active (Q85L) or dominant negative (T39N) Rab32, HA-tagged RTN3L and control pcDNA3. Cells were incubated with Rab32 and RTN3 antibodies. Bar indicates 15 µm. Manders coefficient was calculated for Q85L and T39N. ***p ≤ 0.0001

While Rab32 can show localization to LROs in some cell types, its bulk localizes to the ER in HeLa cells and fibroblasts [3, 26]. From this location, it had been hypothesized that Rab32 could mediate autophagy [13]. Therefore, we decided to test if ER-associated autophagy receptors, including the long version of reticulon-3 (RTN3L), Sec62, FAM134B or CCPG1, would show interaction with active Rab32 as effectors. We chose to analyze their binding to Rab32 by co-immunoprecipitation. We identified RTN3L as a potential Rab32 effector, since it bound preferentially to the Q85L dominant-active mutant (Fig. 3A), while FAM134B binding to Rab32 was non-specific (Fig. 3B). In contrast, the binding of Sec62 to Rab32 was preferentially to the T39N dominant-negative mutant and CCPG1 showed no binding at all (Fig. 3B), providing evidence for the specificity of this interaction. We tested the interaction between Rab32 and RTN3L further by examining interaction between HA-tagged RTN3L and endogenous Rab32, which we could also readily detect (Fig. 3C). To further validate this interaction, we generated N-terminal progressive deletion mutants of RNT3L to gradually remove LC3-interacting region (LIR) motifs, which target autophagy receptors to LC3 associated with phagophore membranes [27]. In the case of RTN3L, there are six LIR motifs, which are also used by Rab9 for recruitment of RTN3L to ER-endosome contacts [28]. We found that removal of two of the most N-terminal LIR motifs with the Δ1–212 mutant of RTN3L already compromised binding of Rab32 to RTN3L, but a further deletion of the next LIR motif in the Δ1–349 mutant virtually abolished binding (Fig. 3D). Together, these results suggested RTN3L could be a Rab32 effector that selectively uses its three N-terminal LIR motifs for this activity.

We investigated this hypothesis by testing whether any of the known ER-phagy receptors shows overlapping distribution with Rab32. To do so, we first performed a biochemical separation of membranes on a Percoll gradient from a MCF7 cell homogenate. On this classic isolation method, Rab32 is known to be part of the mitochondria-associated membranes (MAMs) and peripheral ER microsomes [3]. We were able to confirm this by detecting a nice overlap with the MAM-enriched markers calnexin and TMX1 (Fig. 3E). When analyzing the distribution of known ER-phagy receptors within these fractions, we could not detect a clear-cut overlap of Rab32 with any of them. This could be because endogenous Rab32 corresponds to a mix of active and inactive forms that fractionate to light and heavy ER membranes, respectively (Fig. 3E).

We therefore decided to further analyze the functional interaction between Rab32 and RTN3L via immunofluorescence microscopy, which allowed us to analyze co-localization with both active and inactive Rab32. We first detected overlapping distribution between endogenous Rab32 and endogenous RTN3 (comprising both RTN3S and RTN3L) in HeLa cells on perinuclear structures, consistent with both proteins being largely associated with the ER [3, 29] (Fig. 3F, top). The expression of the long, HA-tagged version of RTN3, HA-RTN3L, showed more staining in the cellular periphery consistent with its fractionation to the microsome fraction in Fig. 3E. This staining did not show significant overlap with endogenous Rab32, but the overlap in the perinuclear area persisted (Fig. 3F, bottom). We next determined the extent of overlap between endogenous RTN3 and active FLAG-tagged Rab32Q85L versus inactive FLAG-tagged Rab32T39N. This comparison showed about 50% more overlap with active Rab32Q85L compared to inactive Rab32T39N (Fig. 3G). Therefore, our findings are consistent with the hypothesis that RTN3L acts as an ER-associated effector of Rab32.

### Active Rab32 triggers degradation of MERC proteins

Our results thus far have identified an autophagy-promoting role of Rab32 as well as an interaction with an ER-phagy receptor, suggesting Rab32-mediated autophagy targets an ER membrane subdomain. To determine the targets of this type of autophagy, we decided to assay for organelle and subdomain-specific substrates in the presence of active Rab32Q85L versus empty plasmid-transfected control cells. Interestingly, we could not detect changes for most candidate substrates in the cytosol, Golgi, endosomes, and mitochondria (Additional file 2: Fig. S2A, B). There were also no decreases for ER marker PDI, the ER sheet marker Climp63, and only slightly higher levels of the ER tubule marker Reticulon-4 (RTN4) (Additional file 2: Fig. S2C). These results suggest Rab32Q85L did not trigger bulk ER-phagy or previously described types of selective ER-phagy [20, 21]. Our results therefore exclude a role for active Rab32 in the autophagic degradation of cytosol, ER, Golgi, and mitochondrial membranes, although some of these are degraded with the help of the Rab32 effector STX17 [24, 30].

**Fig. 4:**
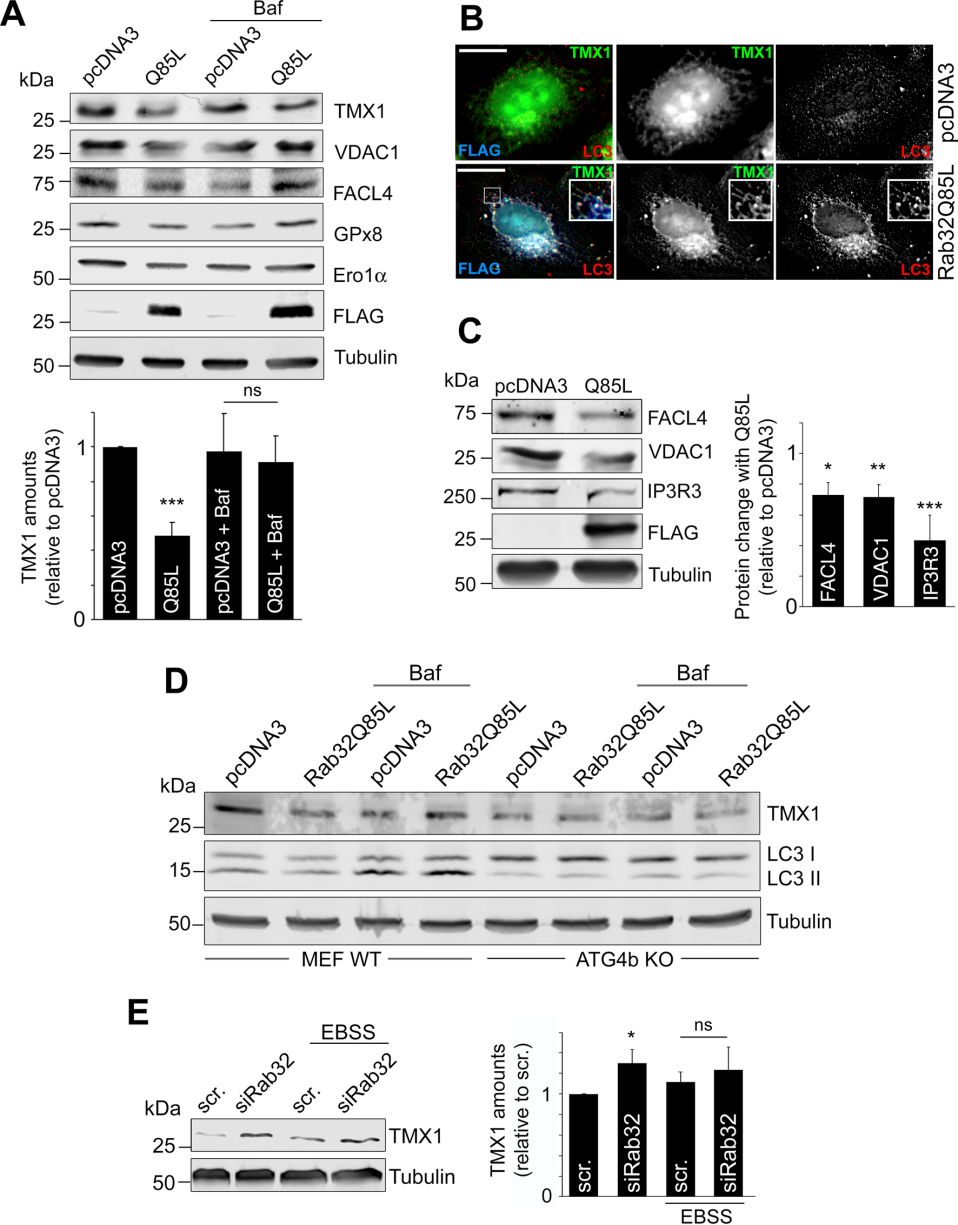
Rab32 Promotes Autophagic Degradation of MAM Proteins. **A** Immunoblot analysis of transfected MCF7 cells with and without 100 nM Bafilomycin A1 incubation for the amounts of MAM proteins. TMX1, VDAC1, FACL4, GPx8 and Ero1α were analyzed. Tubulin acts as a loading control, while FLAG signal indicates transfected Rab32. Densitometry analysis of a minimum n = 3 independent experiments. ***p ≤ 0.0001. **B** Immunofluorescence analysis of TMX1 incorporation into LC3B-decorated structures. Images and insets show control and Rab32Q85L-transfected cells processed for anti-FLAG (blue), anti-LC3B (red) and anti-TMX1 (green) signals. **C** Immunoblot analysis of transfected MCF7 cells for the amounts of additional MERC proteins. FACL4, VDAC1, and IP_3_R3 were analyzed. Tubulin acts as a loading control, while FLAG signal indicates transfected Rab32. n = 3 ***p ≤ 0.0001 **p ≤ 0.01 *p ≤ 0.05. **D** Immunoblot analysis of transfected wild type MEFs and ATG4b knockout MEFs with and without 100 nM Bafilomycin A1 incubation for the amounts of MAM protein TMX1, and LC3. Tubulin was used as loading control. **E** Immunoblot and densitometry analysis of MCF7 cells transfected with control siRNA or siRNA against Rab32, analyzed for MAM protein TMX1 and Tubulin after 4 h EBSS incubation. n = 3, *p ≤ 0.05

Like the closely related Rab29 and Rab38, a portion of Rab32 localizes to MERCs [1]. This prompted us to investigate if proteins localizing to this membrane contact site (MCS) were a target of Rab32-mediated autophagy, including the MERC-enriched TMX1 (Fig. 3E). Our results showed that the activation of Rab32 by Rab32Q85L overexpression for 48 h decreased the amounts of TMX1 by 50% in MCF7 cells (Fig. 4A). Next, we examined whether this reduction derived from autophagic processes with a Bafilomycin A1 block and found that TMX1 no longer showed decreased amounts under these conditions (Fig. 4A). Consistent with TMX1 undergoing autophagic degradation upon activation of Rab32, we noticed extensive co-localization with LC3 in HeLa cells transfected with Rab32Q85L but not in cells transfected with pcDNA3 (Fig. 4B). Similarly, we were able to detect degradation of other MERC-localizing enzymes in MCF7 cells, including glutathione peroxidase 8 (GPx8) and ER oxidoreductin 1 α (Ero1α) [31, 32] (Fig. 4A). Other MERC proteins were also significantly reduced between 60 and 30% with Q85L, including voltage-dependent anion channel 1 (VDAC1) [33], fatty acid coenzyme A ligase 4 (FACL4), and inositol 1,4,5-trisphosphate receptor type 3 (IP_3_R3) [34] (Fig. 4C). In contrast, calnexin did not show such decreases (Additional file 2: Fig. S2C).

The observed decreases for MERC proteins were mitigated in the presence of wild type Rab32 and did not occur upon transfection of dominant-negative Rab32T39N in MCF7 cells (Additional file 2: Fig. S2D). To further investigate whether the degradation of MERC proteins mediated by Rab32 uses the autophagy machinery, we used control and ATG4b knockout mouse embryonic fibroblasts (MEFs) [35]. While we could see autophagy progression and TMX1 degradation in wild type MEFs as triggered by Rab32Q85L, this was not the case in ATG4b knockout MEFs (Fig. 4D).

Next, we wanted to test whether endogenous Rab32 could control the levels of MERC proteins overall. We therefore transfected MCF7 cells with Rab32 RNAi, where we saw that TMX1 increased by about 30% upon Rab32 knockdown for 72 h (Fig. 4E). When starvation-induced autophagy was triggered by incubating cells in EBSS for 2 h, no significant increase was observed (Fig. 4E). Together, our results suggest Rab32 induces a novel type of autophagy that specifically targets MERC proteins.

### Reticulon-3L and Rab32 cooperate to reduce MERCs

**Fig. 5:**
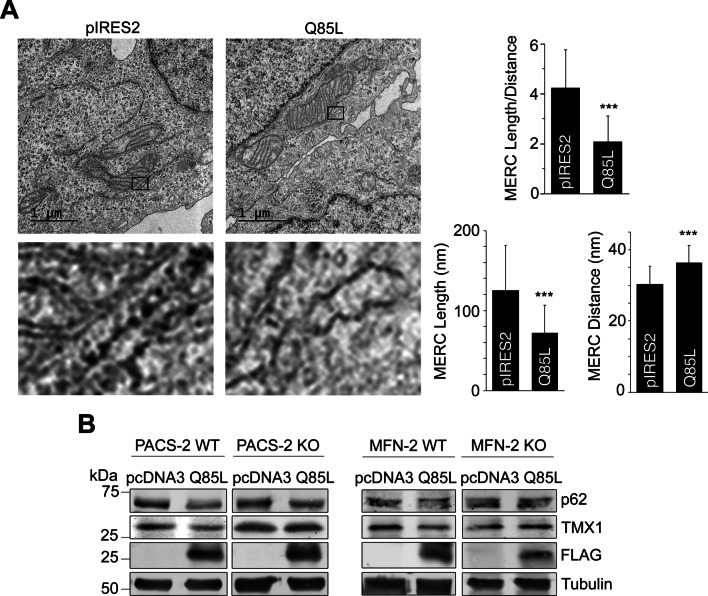
Rab32 Promotes Autophagic Degradation of MERC membranes. **A** Representative images from electron microscopy of stably expressing control pIRES2 or Rab32Q85L cells. Boxes indicate ER-mitochondria contact sites ≤ 50 nm apart. Top: quantification of length of mitochondrial membrane 10-50 nm apart from ER (see below left) divided by average distance of ≤ 50 nm between the two organelle membranes (see below right). n = 100; ***p ≤ 0.0001. Insets show zoomed areas from boxed areas, highlighting typical ER structures in the proximity of mitochondria. **B** Immunoblot of transfected wild type and knockout PACS-2 HeLa and mitofusin-2 mouse embryonic fibroblast (MEF) cells. p62 was probed for comparison and to track Rab32Q85L-induced autophagy

To further investigate this hypothesis, we analyzed the amounts of contacts between the ER and mitochondria via electron micrograph quantifications. These analyses confirmed that in the presence of dominant-active Rab32Q85L, MERCs indeed decrease upon Rab32 activation (Fig. 5A). This reduction in MCS structures was frequently accompanied by an enlargement of ER tubules (see magnifications). Next, we decided to investigate whether ER-mitochondria tethering and RTN3L are required for the Rab32-mediated autophagy mechanism. To do so, we analyzed knockout cells for proteins required for MERC formation. First, we generated Crispr/Cas9-mediated HeLa knockout cells for the MAM tethering regulator phsophofurin acidic cluster sorting protein 2 [36] (PACS-2, Additional file 2: Fig. S2E). We then compared Rab32Q85L-triggered reduction of TMX1 in these cells and in knockout MEFs for the MERC tether mitofusin-2 [37] to their respective wild type counterparts. This analysis showed Rab32Q85L-mediated autophagic degradation of the MAM protein TMX1 did not occur in the absence of PACS-2 or mitofusin-2, suggesting MERC tethering is required for this pathway (Fig. 5B).

**Fig. 6:**
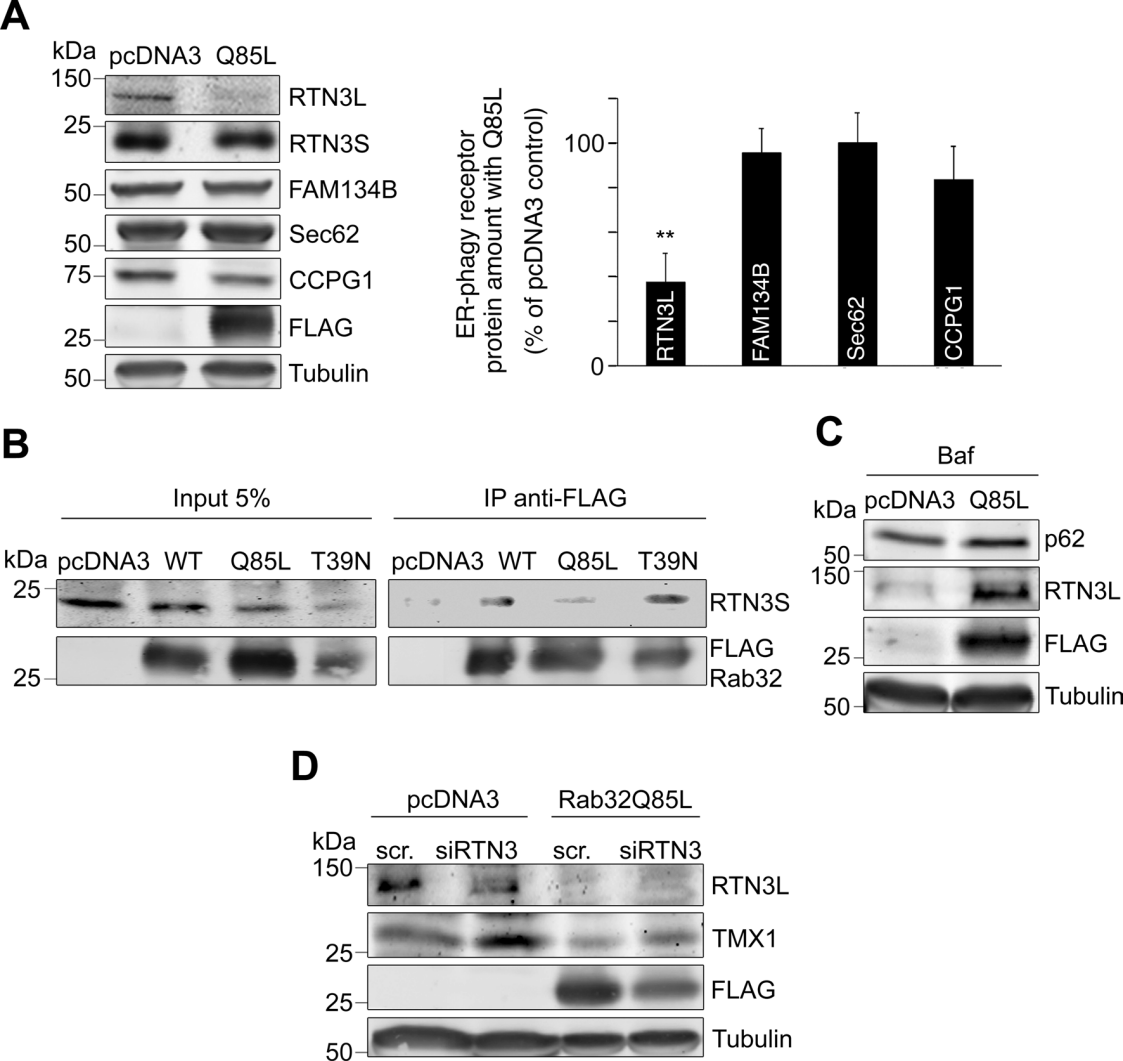
Rab32 cooperates with the RTN3 long form. **A** Immunoblot and densitometry analysis of control pcDNA3 and Q85L-FLAG transfected cells indicates only RTN3L levels decrease with Q85L, while RTN3S, FAM134B, Sec62, and CCPG1 do not show changes. n = 3; **p ≤ 0.01. **B** Co-immunoprecipitation of Rab32 constructs with endogenous RTN3S. MCF7 cells were transfected for 48 h with FLAG-tagged Rab32 constructs, followed by crosslinking, lysis and incubation with anti-FLAG antibodies. Immunoprecipitates were analyzed for anti-FLAG and co-immunoprecipitating endogenous short reticulon-3 (RTN3S). **C** Immunoblot as in A with 100 nM Bafilomycin A1 incubation for 48 h shows accumulation of RTN3L and p62 with Q85L. **D** Immunoblot of MCF7 cells transfected with pcDNA3 and Rab32Q85L co-transfected with control or siRNA against RTN3. RTN3L and FLAG gels assay for the amounts of RTN3L and Rab32. TMX1 amounts were used to assay for autophagy of MAM proteins

Consistent with their reciprocal interaction (Fig. 3A), RTN3L was the only ER-associated autophagy receptor that showed reduced amounts in the presence of Rab32Q85L; a reduction of about 60% (Figs. 3A, 6A) that was of the same order as what we had observed with TMX1 (Fig. 4A). Furthermore, the decrease was specific to the long form of RTN3 and was not observed with short RTN3, which does not have autophagy receptor properties [21] (Fig. 6A). Consistent with no influence of Rab32 on RTN3S, we were also unable to detect interaction with Rab32Q85L (Fig. 6B). As expected, the RTN3L degradation did not occur with Bafilomycin A1 treatment, but rather resulted in RTN3 accumulation under this condition (Fig. 6C). To gain further evidence for a functional connection between Rab32 and RTN3L, we knocked down RTN3 in vector control MCF7 cells and cells transfected with Rab32Q85L. Consistent with Fig. 4A, TMX1 decreases with Rab32Q85L as compared to a pcDNA3 vector control (Fig. 6D, first lane vs. third lane). In contrast, TMX1 levels increased with RTN3 knockdown, suggesting this autophagy receptor normally acts to promote TMX1 degradation (Fig. 6D first lane vs. second lane). In the presence of Rab32Q85L, however, this increase did not reach levels beyond those observed at baseline (Fig. 6D, first lane vs. forth lane). Taking all this information together, our results argue Rab32 regulates autophagy at its origin, rather than at later stages. Moreover, given the narrow substrate specificity of the degradation triggered by Rab32 activity, we propose active Rab32 triggers autophagic degradation of MERCs and, hence, MAMs, in a process we propose to call “MAM-phagy”.

## Discussion

**Fig. 7:**
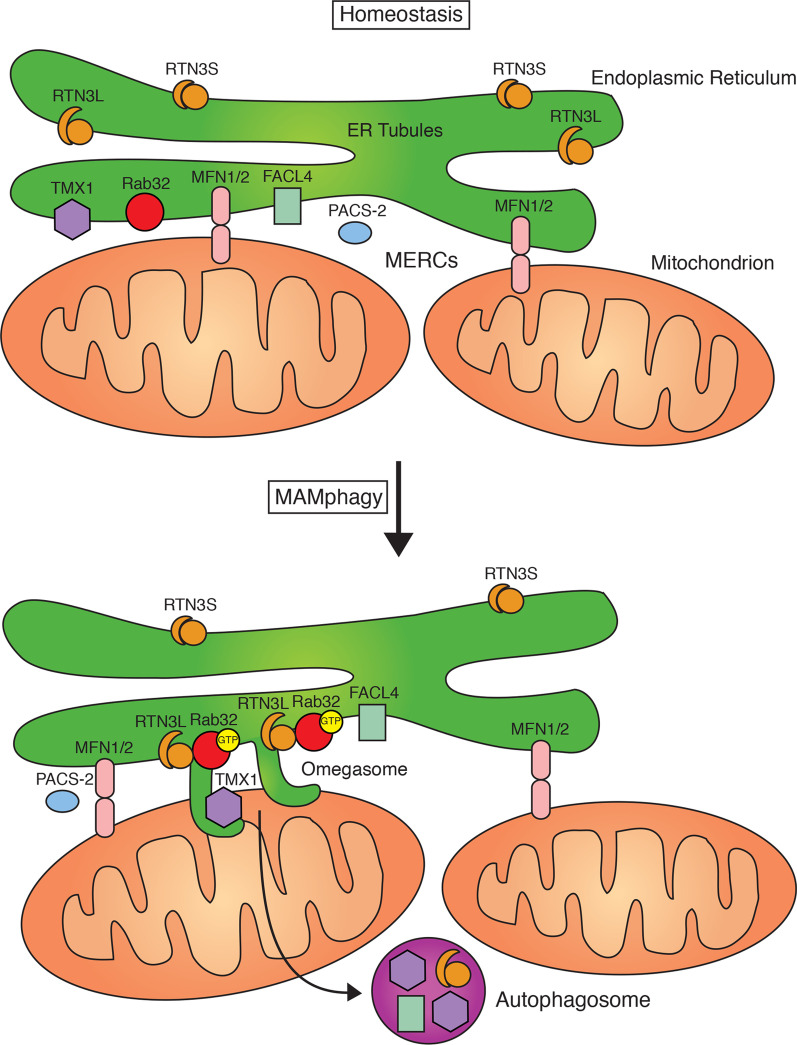
Model for Rab32-Mediated MAM-phagy. Activation of Rab32 triggers its interaction with the RTN3L effector. This promotes the selective autophagy of MERC-localized proteins, biochemically isolated as MAMs, and results in the degradation of TMX1, FACL4, and RTN3L itself. Rab32-mediated autophagy of MAMs requires the presence of PACS-2 and Mfn2

Research on the origin of autophagosomal membranes have identified MERCs as prominent sources for this mechanism [23]. Upon disruption of the proteinaceous tethering between the two organelles, this source material for autophagy becomes less abundant. These observations suggest that an autophagy-associated tubulation or vesiculation pathway exists that requires MERCs formation. Such a pathway apparently depends on the activity of PACS-2, one of the first described factors that promote MERC formation and enrichment of ER proteins within MAM biochemical fractions [23, 36]. Another requirement for this type of selective autophagy is the presence of mitofusin-2 [38] (Fig. 7). Importantly, mitophagy proceeds even in the absence of mitofusin-2 [39], confirming the distinction between the two types of selective autophagy, targeting mitochondria or MERCs, respectively. A role for mitofusin-2 in energy–stress induced autophagy that controls MERC formation has recently been confirmed [40]. However, to date, no membrane-shaping or membrane-vesiculating machinery has been described that could control this selective autophagy pathway. We hypothesized Rab32 could be part of such a machinery, because it is a known autophagy inducer [13], but also extracts MERC proteins and shuttles them to the cellular periphery [3]. When investigating this mechanism, we identified RTN3L as a Rab32 effector. Interestingly, this ER-shaping protein has recently also been found to control ER-endosome membrane contacts and controls their formation during the maturation of endosomes in a Rab9-dependent manner [28]. The synthesis of those results and our study suggests that Rab32 could cooperate with Rab9 in a Rab activation cascade [41].

In the results presented in this manuscript, we identify an autophagic mechanism that is accelerated in the presence of active Rab32 but inhibited upon Rab32 knockdown. Given its narrow substrate specificity that is limited to MERC proteins, our result strongly suggest Rab32 regulates autophagy at its origin rather than at later stages. Our results also demonstrate that the two other Rab32 family proteins, Rab38 and Rab29, are not equipped with such a function, which lends further evidence to the hypothesis that Rab29 executes a unique non-redundant, as we had previously speculated [1]. Further research will have to address this possibility.

Interestingly, the susceptibility of MERC proteins to Rab32-mediated autophagy varied. While TMX1 consistently showed autophagic degradation in the presence of active Rab32Q85L, we never detected decreases of calnexin, which also significantly associates with MERCs. A potential explanation for this difference could be the distinct MERC targeting between calnexin and TMX1, where TMX1 is associated with detergent-resistant membranes while calnexin is not [42]. Previous research has also shown calnexin preferentially associates with core autophagy initiators such as AMBRA1 [43]. Such autophagy initiators are removed from the isolation membrane before closure of the autophagosome and are therefore not degraded. Therefore, our results are consistent with calnexin recruiting autophagy initiators and not being incorporated into the autophagosome itself. Another role for calnexin in autophagy includes its recruitment of ER-phagy receptor FAM134B during the degradation of ER sheets [44]. This is further evidence that the FAM134B autophagy receptor and calnexin are not directly involved in the type of selective autophagy of MERCs described here. Moreover, global activation of RTN3L-mediated ER-phagy triggers calnexin degradation [21], again suggesting the more selective autophagy mediated by the Rab32-RTN3L interaction we describe here is distinct. In contrast to the Rab32-mediated subtype of autophagy, RTN3L-associated global ER-phagy causes the degradation of Reticulon-4, which however resisted Rab32Q85L-mediated degradation (Additional file 2: Fig. S2C). Therefore, RTN3L might promote distinct ER remodeling and autophagic pathways, dependent on the upstream activating GTPase, which in the case of MAM-phagy is Rab32.

## Conclusions

The results presented in this manuscript describe, for the first time, a detailed mechanism for the role of Rab32 in autophagy by identifying an autophagy-related effector as well as the target membrane of its autophagic activity. In brief, our results show RTN3L acts as an effector molecule for active Rab32, which promotes the selective autophagy of MERC-localized proteins, biochemically isolated as MAMs (Fig. 7). This cell biological role in MAM-phagy distinguishes Rab32 from its closely related Rab family members Rab38 and Rab29 but could associate it with endosomal Rab9.

## Materials and methods

### Antibodies, reagents and plasmids

Antibodies for Rab32 (Sigma HPA025731), FLAG (Rockland 600-401-383 for Western blot and 200-301-B13 for microscopy), HA (Cell Signaling, rabbit monoclonal #3724), HA.11 (Biolegend 901501), VDAC1 (ab14734 Abcam), Complex II (ab14714 Abcam), Drp1 (ab56788 Abcam), FAM134b (ab151755), p62 (610833 BD BioSciences), BiP (610978 BD Biosciences), Tubulin (T 5168 Sigma), Sec62 (HPA014059 Sigma), Syntaxin-17 (HPA001204 Sigma), LC3 (#12741 for Western blot and #2775 for microscopy, Cell Signaling), GAPDH (#5174, Cell Signaling), Reticulon-3 (Thermo MA5-15538 for microscopy and Boster Biological PA2256 for Western blot), Reticulon-4 (10740-1-AP ProteinTech), PACS-2 (19508-1-AP ProteinTech), CCPG1 (13861-1-AP ProteinTech), Golgin 97 (A-21270 Molecular Probes), Complex IV-sub IV (20E8C12 Life technologies), PDI (MA3-019 Thermo), Climp 63 (C5840 US Biological), Sec61B (07-205 Millipore), IP_3_R3 (BD Biosciences 610312), FACL4 (Abcam ab11007), and TMX1 (Abnova H008154-B01P for Western blot and Thermo MA5-26309) were purchased as indicated. Anti-EEA1, and anti-Cytochrome C were kind gifts from Paul Melancon and Michele Berry, respectively. The polyclonal calnexin antibodies were described previously [3]. Rab29, Rab38, and Rab32 plasmids and the Rab32 serum antibody were described previously [1, 26]. Human Rab32 siRNA (HSS116975), Reticulon-3 (HSS145573), and control STEALTH siRNA (452001) were from Invitrogen. EBSS (Thermo Fisher), DAPI (Sigma), Bafilomycin A1 (Millipore), and Percoll (GE Healthcare) were purchased as indicated.

RTN3L-expressing plasmids were generated by Precision Biolaboratories (St. Albert, AB) from a 3HA-tagged template (Genecopoiea) using the following oligos and inserted into pCEP4, START/STOP codons in bold and restriction site underlined: 3′ (with Xho I cloning site): CTTGCCGGCCTCGAGCTATTCTGCCTTT; 5′ Δ1-212 AGGAGTTCGAACC**ATG**GATGACAGATTCACTTTGCTGACAGC; 5′ Δ1-349 AGGAGTTCGAACC**ATG**ATACTGACTTGGGATCTGGTTCCCCAAG; 5′ D1-562 AGGAGTTCGAACC**ATG**TCTAAAAACTTTGAAGAATTGGTCAGTG.

### Cell culture and transfection

MCF7 and MCF7-LC3-GFP were from ATCC and a kind gift from Ing-Swie Goping. Wild type MEFs and ATG4b knockout MEFs were a kind gift from Guillermo Marino Garcia (University of Oviedo, Spain). MCF7 were cultured in RPMI (12633012) and MCF7-LC3-GFP in RPMI supplemented with 50 mg/mL Geneticin (10131027), both with 10% FBS (12483020), all from Gibco. MFN-2 WT and KO MEFs were from David Chan (California Institute of Technology, Pasadena), both were cultured with DMEM (Gibco) and 10% FBS. MCF7 were transfected with lipofectamine 2000 (Invitrogen) or with Oligofectamine for siRNA (Invitrogen) and HeLas and MEFs with Metafectene (Biontex). For immunofluorescence, MCF7 were nucleofected with the Lonza nucleofector kit V in their Nucleofector 2b device with protocol P-020. The Hela PACS-2 knockout cell line (clone 129) was generated using Crispr/Cas9 methods based on the following sgRNA primer pair. Left: ACAGCTGGGGTGATGTCTTG, right: CTTGCCCCAGGAACTTCCAG (PMID 24157548).

### Immunofluorescence microscopy

MCF7 were nucleofected as described above and grown on coverslips. RPMI was replaced with EBSS for 2 h where indicated. For GFP-LC3 quantification, cells were then washed with PBS++ (PBS with 1 mM CaCl_2_ and 0.5 mM MgCl_2_) and fixed with 4% paraformaldehyde (Sigma) for 20 min. Cells were then washed again with PBS++ and permeabilized with 0.1% Triton X-100 + 0.2% BSA in PBS++ for 2 min. Cells were incubated with DAPI for 5 min, washed and incubated with primary antibodies (1:100) for 1 h. Cells were washed and incubated with secondary antibodies (1:1000) (AlexaFluor-conjugated 488 or 594 from Invitrogen) for 30 min and mounted in Prolong AntiFade (Invitrogen). For co-localization, HeLa cells transfected as indicated were fixed in 3% paraformaldehyde (Sigma) + 0.1% glutaraldehyde (Sigma) in PBS++ for 20 min. Cells were permeabilized with 0.1% Triton X-100 + 0.2% BSA in PBS++ for 1 min. Cells were blocked for 15 min in 0.2% Saponin + 2% BSA in PBS++. Primary antibodies (mouse anti-TMX1, 1:100; rabbit anti-LC3B, 1:100; RTN3, 1:100; rabbit anti-Rab32, 1:50; mouse anti-Flag, 1:250; mouse anti-HA clone HA.11, 1:200) were added for 1 h in the same buffer, while secondary antibody incubation (Alexa Fluor 488 goat anti mouse, Invitrogen A11029, 1:1000; Alexa Fluor 594 goat anti rabbit, Invitrogen A11012, 1:1000) was for 30 min. Cells were washed in 0.2% Saponin + 0.2% BSA in PBS++ and mounted as above. Coverslips were imaged with an Axiocam on an Axio Observer microscope (Carl Zeiss, Jena, Germany) using a 63X plan-Apochromat lens. Iterative deconvolution was performed with the Axiovision software and images enhanced with Photoshop (Adobe, San Jose, CA) using the levels function only to achieve near-saturation levels in the brightest areas of each channel. For the LC3 puncta assay, ImageJ was used to annotate images to facilitate counting cells and their LC3 staining pattern. Cells with ≥ 10 LC3 puncta were considered to have higher than basal level of autophagy. Samples were analyzed in a blinded manner. ≥ 50 cells per condition were counted in each experiment. For the co-localization analysis, Manders coefficients were calculated with ImageJ.

### Western blotting

Western blots were done following standard protocols with secondary antibodies (1:10,000) conjugated to Alexafluor 488 or 594 (Invitrogen) detected on an Odyssey infrared imaging system (LICOR, Lincoln, NE). Quantification was performed with ImageStudio Lite.

### Co-immunoprecipitations

Cells were washed with PBS++ and crosslinked with 2 mM 3,3'-Dithiodipropionic acid di(N-hydroxysuccinimide ester (DSP, Thermo) for 30 min at room temperature. Cells were then washed with NH_4_Cl in PBS++ to quench the cross-linking reaction, harvested in Chaps buffer (150 mM NaCl, 10 mM Tris pH 7.4, 1 mM EDTA, 1% Chaps) and centrifuged at 800 rpm for 10 min at 4 °C. The supernatant was precleared by incubating with PAS beads for 1 h on a rocker at 4 °C. The samples were then centrifuged at 800 rpm for 2 min and the supernatant was incubated similarly with 5 mL of anti-FLAG antibody overnight. 25 mL of Protein A Sepharose (PAS) beads (GE Healthcare) were added for protein precipitation for 1 h on a rocker at 4 °C the next day and then samples were washed with Chaps buffer before being analyzed by western blot.

### Percoll fractionation

MCF7 cells cultured in 15 15 cm dishes, washed with PBS++, harvested with 4 mL of mitochondrial homogenization buffer (10 mM HEPES, pH 7.4, 250 mM sucrose, 1 mM EDTA, and 1 mM EGTA) and centrifuged 5 min at 1,500 rpm in a JA-12 rotor at 4 °C. The pellet was resuspended in 5 mL of buffer and passaged 8 times back and forth through a ball bearing homogenizer with a 14 μm ball. The homogenate was then centrifuged twice at 1,810 rpm for 10 min in a JA-12 rotor to remove nuclei and debris. A sample of supernatant was taken and used as the homogenate fraction while remaining supernatant was spun at 8,500 rpm for 10 min in a JA-12 rotor. The resulting supernatant was separated into several Eppendorf 1.5 ml tubes and precipitated with acetone overnight to obtain the cytosolic fraction while the pellet was resuspended in 1 mL of buffer. A small aliquot was taken as the crude mitochondria fraction, and the remaining layered onto an 18% Percoll solution in a polycarbonate tube. Samples were then centrifuged at 33,333 rpm for 30 min in a 90Ti rotor. The MAM fraction was isolated roughly ¾ down the tube and the pure mitochondria fraction underneath the MAM fraction. To remove Percoll, the MAM fraction was centrifuged at 60,000 rpm for 1 h in a TLA 120.2 rotor. The pure mitochondrial fraction was also cleared of Percoll by transferring to 4 Eppendorf tubes and homogenization buffer was added before centrifuging twice at 10,000 rcf for 10 min, removing supernatant and adding fresh buffer in between spins. Equal proportional amounts of each fraction were analyzed by immunoblot.

### Electron microscopy

Cells were fixed with filtered 2% paraformaldehyde and 2% glutaraldehyde in 0.1 M sodium cacodylate buffer, pH 7.4, for 30 min at 37 °C. Cells were scraped with sodium cacodylate buffer and pelleted in a series of increasing centrifugation speeds, from 1000 to 12,000 g. Post fixation was done with 1% osmium tetroxide in the sodium cacodylate buffer for 1 h on ice. Samples were quickly rinsed twice with water before staining with 1% aqueous uranyl acetate 18 h in a rocker protected from light. Dehydration was done with increasing concentrations of ethanol and infiltration with two 10 min incubations of acetonitril. Samples were then covered with a 1:1 mixture of acetonitril and Embed 812 and placed under vacuum for 18 h. Lastly, samples were placed under vacuum for 5 h with pure Embed 812 and then allowed to harden at 60 °C for 48 h before sectioning. We used an Ultracut E (Reichert-Jung) for sectioning and imaged the samples using a CCD camera (iTEM; Olympus Soft Imaging Solutions) mounted on a 410 transmission electron microscope (Philips). Images were analyzed for ER tubules in close proximity to mitochondria and quantified with ImageJ. Distances between the organelles were all kept under 50 nm to be considered a membrane contact site. Data was derived from over 50 images per condition with n = 100 measurements each.

### Statistical analysis

All the data are expressed as mean ± standard deviation. Bar graphs include standard errors. Statistical significance was determined with student’s unpaired *t-*test using QuickCalcs by GraphPad (https://www.graphpad.com/quickcalcs/ttest1/).

## Supplementary Information

**Additional file 1: Fig. S1.** Rab32 family members Rab38 and Rab29 do not induce autophagy. **A.** Representative immunofluorescence images of MCF7 cells stably expressing GFP-LC3 after a transfection with FLAG-tagged dominant active Rab32, Rab38, and Rab29. FLAG negative cells were used as a control. Bar indicates 15 µm. Quantification of cells with > 10 LC3 puncta, indicating increased autophagy, were expressed as a percent of total cells counted. ≥ 50 cells per condition were counted in each (n = 3) independent experiment **p ≤ 0.001. **B.** Immunoblot of MCF7 cells transfected with control pcDNA3 or FLAG-tagged dominant active (Q85L). Cells were treated with Bafilomycin A1 for 48 h where indicated. Rab32Q85L induces a small decrease in p62 that is rescued with Bafilomycin A1. **C.** Representative immunofluorescence images after a 2 h incubation with EBSS of MCF7 cells stably expressing GFP-LC3 and transfected with FLAG-tagged wild type (WT), dominant active (Q85L) or dominant negative (T39N) Rab32, probed for FLAG-tagged Rab32 (red) and nuclei (DAPI, blue) in parallel. Bar indicates 15 µm. Quantification of cells with > 10 LC3 puncta, indicating increased autophagy, were expressed as a percent of total cells counted. ≥ 100 cells per condition were counted in each (n = 3) independent experiment; all *p* > 0.05. **D.** Immunoblot and densitometry analysis of MCF7 cells transfected with FLAG-tagged wild type (WT), dominant active (Q85L) or dominant negative (T39N) Rab32, analyzed for LC3II/Tubulin after a 4 h EBSS or 48 h 100 nM Bafilomycin A1 incubation. n = 3; ***p ≤ 0.0001.

**Additional file 2: Fig. S2.** Only dominant active Rab32 targets MERCs and their proteins for autophagic degradation. **A.** Immunoblot analysis of transfected MCF7 cells for the amounts of cytosolic, endosomal and Golgi proteins. GAPDH, EEA1, Syntaxin 17, and Golgin97 were analyzed. Tubulin acts as a loading control, while FLAG signal indicates transfected Rab32. **B.** Immunoblot analysis of transfected MCF7 cells for the amounts of mitochondrial proteins. Mitochondrial complex II, cytochrome c, complex IV subunit IV, mitofusin-1 and Drp-1 were analyzed. Tubulin acts as a loading control, while FLAG signal indicates transfected Rab32. **C.** Immunoblot analysis of transfected MCF7 cells for the amounts of ER proteins. PDI, BiP, Calnexin, Climp63, Reticulon-4 and Sec61B were analyzed. Tubulin acts as a loading control, while FLAG signal indicates transfected Rab32. **D.** Immunoblot for MCF7 cells transfected with control pcDNA3 or WT, T39N, and Q85L-FLAG tagged Rab32 shows only Q85L efficiently degrades MAM proteins TMX1, and VDAC1, while ER (calnexin, BiP), mitochondrial (Drp1, complex IV subunit IV) and cytosolic markers (GAPDH) remain unaffected. **E.** Immunoblot analysis of parental HeLa cells and Crispr/Cas9-generated PACS-2 knockout cells. Lysates were quantified for equal protein content and analyzed by Western blot for PACS-2, using actin as a loading control.

## Data Availability

All data and material are available from the corresponding author.

## Abbreviations

AMBRA1: Autophagy and beclin1 regulator 1
BLOC-3: Biogenesis of lysosome-related organelles complex 3
CCPG1: Cell cycle progression 1
Drp1: Dynamin-related protein 1
EBSS: Earle’s balanced salt solution
ER: Endoplasmic reticulum
FACL4: Fatty acid coenzyme A ligase 4
FAM134B: Family with sequence similarity 134 member B
GFP: Green fluorescent protein
GPx8: Glutathione peroxidase 8
KO: Knockout
LRO: Lysosome-related organelle
LRRK2: Leucin-rich repeat kinase 2
MAM: Mitochondria-associated membrane
MEF: Mouse embryonic fibroblast
MERC: Mitochondria-ER contacts
mPAS: Mammalian pre-autophagosomal structure
mTORC1: Mammalian target of rapamycin complex 1
NDP52: Nuclear dot protein of 52 kDa
PACS-2: Phosphofurin acidic cluster sorting protein 2
PBS: Phosphate-buffered saline
PKA: CAMP-dependent protein kinase A
RTN3: Reticulon 3
SNX6: Sorting nexin 6
STX17: Syntaxin 17
TBC1D14: TBC-domain family member 14
TMX1: Thioredoxin transmembrane domain protein 1
UPR: Unfolded protein response
VDAC1: Voltage-dependent anion channel 1
WT: Wild type
